# Effect of Co Doping on the Physical Properties and Organic Pollutant Photodegradation Efficiency of ZnO Nanoparticles for Environmental Applications

**DOI:** 10.3390/nano14010122

**Published:** 2024-01-04

**Authors:** Hajer Saadi, Othmen Khaldi, João Pina, Telma Costa, J. Sérgio Seixas de Melo, Paula Vilarinho, Zohra Benzarti

**Affiliations:** 1 Laboratory of Multifunctional Materials and Applications (LaMMA), Department of Physics, Faculty of Sciences of Sfax, University of Sfax, Soukra Road km 3.5, B.P. 1171, Sfax 3000, Tunisia; hajermosbahsaadi@gmail.com; 2 LMOP(LR99ES17), Faculty of Sciences of Tunis, University of Tunis El Manar, Tunis 2092, Tunisia; othmen.khaldi@gmail.com; 3 CQC-IMS, Department of Chemistry, University of Coimbra, 3004-535 Coimbra, Portugal; jpina@qui.uc.pt (J.P.); tcosta@qui.uc.pt (T.C.);; 4 CICECO–Aveiro Institute of Materials, Department of Materials and Ceramic Engineering, University of Aveiro, 3810-193 Aveiro, Portugal; paula.vilarinho@ua.pt; 5 CEMMPRE, ARISE, Department of Mechanical Engineering, University of Coimbra, Rua Luís Reis Santos, 3030-788 Coimbra, Portugal

**Keywords:** Co-doped ZnO, DFT calculations, Raman spectroscopy, photocatalytic properties

## Abstract

This paper presents a comprehensive investigation of the synthesis and characterization of Zn_1−x_Co_x_O (0 ≤ x ≤ 0.05) nanopowders using a chemical co-precipitation approach. The structural, morphological, and vibrational properties of the resulting ZnO nanostructures were assessed through X-ray diffraction, scanning electronic microscopy, and Raman spectroscopy to examine the influence of cobalt doping. Remarkably, a notable congruence between the experimental results and the density functional theory (DFT) calculations for the Co-doped ZnO system was achieved. Structural analysis revealed well-crystallized hexagonal wurtzite structures across all samples. The SEM images demonstrated the formation of spherical nanoparticles in all the samples. The vibrational properties confirmed the formation of a hexagonal wurtzite structure, with an additional Raman peak corresponding to the F_2g_ vibrational mode characteristic of the secondary phase of ZnCo_2_O_4_ observed at a 5% cobalt doping concentration. Furthermore, a theoretical examination of cobalt doping’s impact on the elastic properties of ZnO demonstrated enhanced mechanical behavior, which improves stability, recyclability, and photocatalytic activity. The photocatalytic study of the synthesized compositions for methylene blue (MB) dye degradation over 100 min of UV light irradiation demonstrated that Co doping significantly improves photocatalytic degradation. The material’s prolonged lifetime, reduced rate of photogenerated charge carrier recombination, and increased surface area were identified as pivotal factors accelerating the degradation process. Notably, the photocatalyst with a Zn_0.99_Co_0.01_O composition exhibited exceptional efficiency compared to that reported in the literature. It demonstrated high removal activity, achieving an efficiency of about 97% in a shorter degradation time. This study underscores the structural and photocatalytic advancements in the ZnO system, particularly at lower cobalt doping concentrations (1%). The developed photocatalyst exhibits promise for environmental applications owing to its superior photocatalytic performance.

## 1. Introduction

In recent times, significant efforts have been directed towards the synthesis of semiconductors with photocatalytic features owing to their potential to address crucial environmental issues. Among these concerns, water pollution stands out as a global challenge, one exacerbated by dye effluents and dye industries [1]. Unfortunately, these classic methods are expensive and cannot completely degrade organic pollutants. In response, various conventional wastewater treatment methods, such as oxidation, reduction, anaerobic, aerobic, electrochemical, biological, and precipitation treatments, have been extensively employed [2,3,4,5,6,7]. However, these conventional approaches tend to be costly and often fall short of completely degrading organic pollutants, resulting in the release of residual liquids containing dyes that contribute to environmental pollution [8]. 

In this context, the photodegradation of organic pollutants with semiconductor metal oxides has emerged as a promising solution for water purification. This approach is characterized by its efficacy in degrading a wide range of nondegradable and toxic organic pollutants in wastewater without necessitating complex technologies [9,10]. Zinc oxide (ZnO) semiconductors have garnered considerable attention for their potential application in environmental protection and have been extensively studied for their versatile structural, optical, electronic, piezoelectric, and transparent conducting properties [11,12]. ZnO nanostructures, in particular, have gained prominence due to their ease of fabrication, cost-effectiveness, non-toxicity, excellent chemical and mechanical stabilities, environmental friendliness, and abundant natural availability [13]. Zinc oxide is considered a multifunctional material owing to its exceptional physical behavior. 

Structurally, ZnO is a II–IV semiconductor compound that crystallizes in a hexagonal wurtzite phase, known for its stability under normal conditions. It displays n-type conductivity with a considerable direct optical band gap energy of roughly 3.37 eV coupled with an elevated exciton binding energy of 60 meV at 300 K [14]. It is also characterized by its good carrier mobility and long shelf-life, enhanced transparency in the visible domain, large electro-optic coefficient, elevated piezoelectric constant, and susceptibility to the doping effect [15]. Some researchers have extensively utilized ZnO in the photocatalytic degradation of organic pollutants [16]. When excited, ZnO generates electron–hole pairs in its photocatalyst conduction and valence bands, respectively. These photogenerated charge carriers react with surface molecules (such as H_2_O and adsorbed O_2_) to undergo secondary chemical reactions that produce radical species (OH· and O^2−^), which in turn react with the organic pollutants to degrade them into undamaging by-products, including H_2_O and CO_2_ [9,17]. 

However, ZnO’s photocatalytic performance is hindered by its limited ability to absorb visible light and its rapid recombination of electron–hole pairs, making it less suitable for commercial applications in contaminant degradation [18]. Moreover, its efficiency is compromised due to the short lifetimes (0.322 ns) of the photogenerated carriers, leading to increased recombination during the photocatalysis process [19]. The efficiency of photocatalysis is, unfortunately, heavily reliant on the effective transfer and separation of photogenerated charge carriers. Hence, suppressing the recombination of the photoinduced carriers is crucial for enhancing the performance of ZnO photocatalyst systems. To address these challenges, various strategies have been employed to improve the photocatalytic efficiency of ZnO, namely modifying and controlling structural and textural characteristics such as size, shape, and porosity [3]. It is essential to manipulate these features through the doping/Co doping effect, the deposition of metal materials on the ZnO surface to create a heterojunction at the interface [8], and merging with another semiconductor [20]. 

For this purpose, numerous studies have reported that the doping of various transition metal elements such as Fe [21], Cu [22,23], Mn [24], and Ni [25] offers a feasible means of enhancing the physical properties, especially the photocatalytic efficiency, of ZnO. Among the various cationic dopants, cobalt is considered to be one of the most effective species to tune both electronic and optical properties due to its abundant electronic states as well as its minor influence on the ZnO lattice structure. Thanks to its non-toxic nature and ready availability, Co metal is a good choice as a dopant to improve photocatalytic activity efficiency. In fact, the introduction of Co in the ZnO host lattice creates dopant energy levels located between the valence band and conduction band of ZnO. Consequently, the photogenerated carriers are trapped at these localized positions, resulting in a decrease in the recombination rate, which ultimately enhances the photocatalytic activity.

For instance, Zhang et al. [26] investigated the photocatalytic efficiency of Co doped hydrothermally ZnO nanoarray, reporting improvements in photocatalytic performance upon Co addition and its significant contribution to degrading the organic pollution in the water environment. Sutka et al. [27] explored the impact of Co doping in the improvement of photocatalytic characteristics of ZnO nanowires prepared using a solvothermal process. Several studies have emphasized that effective photocatalysts should exhibit, among them, (i) a strong affinity of the pollutant molecule to facilitate electron exchange, (ii) a reduced recombination rate of excited electrons to offer adequate time for the degradation of organic molecules, ultimately achieving the best photocatalytic performance for ZnO system [17]. Other factors such as the purity of the products, size, morphology of the nanoparticles, and specific surface area are the main ones responsible for changing the properties of metal oxide and especially the photocatalytic response [28,29]. Essentially, the recombination process is closely linked to nanoparticle size, with the largest particle size having a lower recombination probability, increasing photocatalytic activity [30]. 

In this context, different explanations were proposed for the enhancement of the photocatalytic activity of the metal oxide system. Several groups have demonstrated that high crystallinity and large specific surface areas are responsible for high photocatalytic activity in terms of the degradation of pollutants [31]. By contrast, other authors have revealed that photocatalytic properties increase with an increase in the particle size and a decrease in the specific large surface area [15,30].

Several factors, including the solution pH, dye nature, initial dye and catalyst concentrations, and surface defects, can strongly influence degradation efficiency [32]. Moreover, the performance of the photodegradation response is also contingent on the exciton lifetime, which is a crucial indicator of material quality and radiative recombination efficiency.

Undeniably, the method of preparation strongly influences the photocatalytic activity of the material. Various chemical and physical techniques have been employed to produce ZnO nanostructures, including the sol–gel method [33,34], hydrothermal process [1,11], spray-pyrolysis [35], solid-state reaction technique [36], and the microwave method [37].

Notably, the co-precipitation method is widely favored due to its simplicity, high purity, low operating temperature, reduced surface imperfection, superior uniformity and purity of the resulting products, and ease of checking the size and the change in the morphology that can intensely modify the physical features of the prepared compound [38].

A survey of the literature reveals the enhancement of the photocatalytic properties of ZnO nanostructures, employing various preparation methods. However, the improvement of this property under the Co doping effect adopted, particularly by the chemical co-precipitation approach and employing zinc chloride ZnCl_2_·6H_2_O and cobalt chloride CoCl_2_·6H_2_O, is scarce and still to be furnished. On the other hand, there exists a lack of prior theoretical investigation on the impact of Co introduction into ZnO nanoparticles. Therefore, in this study, Zn_1−x_Co_x_O (0 ≤ x ≤ 0.05) nanoparticles were synthesized using the co-precipitation method, and the effect of Co dopant on their structural, morphological, mechanical, and photocatalytic properties was studied and discussed in detail. This research offers, for the first time, a comprehensive examination of physical properties, considering both experimental and theoretical studies. The enhancement of the structural, mechanical, and photocatalytic properties of ZnO nanoparticles under Co doping makes these compositions promising candidates for environmental applications.

## 2. Materials and Methods

### 2.1. Computational Method

First-principles calculations were performed using density functional theory (DFT) with the Becke Lee–Yang Parr (B3LYP) method, which was implemented in the CRYSTAL17 v.1.0.2 software package. To represent crystalline orbitals as a linear combination of Bloch functions, an all-electron basis set comprising Gaussian-type functions was utilized for both oxygen and zinc. For oxygen, a [4s3p] basis set, along with an additional d orbital (with an exponent of 0.5), was employed, resulting in a [4s3p1d] basis set. For zinc, a [6s5p2d] basis set was used. 

The geometries were optimized based on the convergence of analytical gradients and nuclear displacements. The diagonalization process involved the use of a grid of k points, following the Monkhorst–Pack method. The shrinking factor was set to 8 × 8 × 8, which corresponds to 50 independent k points in the Brillouin zone. An analytical approach was employed to simulate the relative intensities of vibrational peaks of the IR and Raman properties. This approach combines gradients of mono-electronic and bi-electronic integrals. The formalism utilizes a coupled perturbed Hartree–Fock/Kohn–Sham scheme to analyze the response of crystalline orbitals to a static electric field. In the simulations, a convergence threshold of 10^−8^ Hartree was set for the self-consistent-field (SCF) procedure during structural optimizations. For vibration frequency calculations, a more stringent convergence threshold of 10^−10^ Hartree was employed.

### 2.2. Synthesis of ZnO and Cobalt-Doped ZnO Nanopowders

The synthesis of ZnO nanopowders included zinc chloride ZnCl_2_·6H_2_O with a purity of ≥99.995% from Sigma–Aldrich and cobalt chloride CoCl_2_·6H_2_O with a purity of ≥97% from Sigma–Aldrich. Methylene blue (MB) was chosen as the model organic pollutant for this study. The synthesis of both ZnO and cobalt-doped ZnO nanopowders involved the use of ethanol and distilled water. 

For this experiment, zinc chloride and cobalt chloride were selected as the suitable precursors in the co-precipitation method used to synthesize three compositions of Zn_1−x_Co_x_O with different cobalt doping concentrations (x = 0, 0.01, and 0.05). For the preparation of the ZnO sample, the following procedure was followed: First, 3.5 g of zinc chloride was dissolved in 50 mL of distilled water under magnetic stirring at 300 K for 15 min. Second, a stoichiometry amount of 4 g of NaOH was separately prepared and fully dissolved in 100 mL of deionized water. Third, 10 mL of the obtained solution was added at 15-min intervals to the zinc chloride solution. The resulting mixture solution was magnetically stirred for 3 h to get a white precipitate. The latter was then thoroughly washed several times with distilled water to remove any remaining residues and impurities. Finally, it was oven-dried at a temperature of 80 °C for 12 h, complemented by calcination for 5 h at the temperature of 500 °C. 

The same protocol was applied for the preparation of Co-doped ZnO nanoparticles by introducing 3.318 g of ZnCl_2_·6H_2_O and 0.031 g of CoCl_2_·6H_2_O for Zn_0.99_Co_0.01_O sample and 3.148 g of ZnCl_2_·6H_2_O and 0.159 g of CoCl_2_·6H_2_O for Zn_0.95_Co_0.05_O sample.

**Figure 1:**
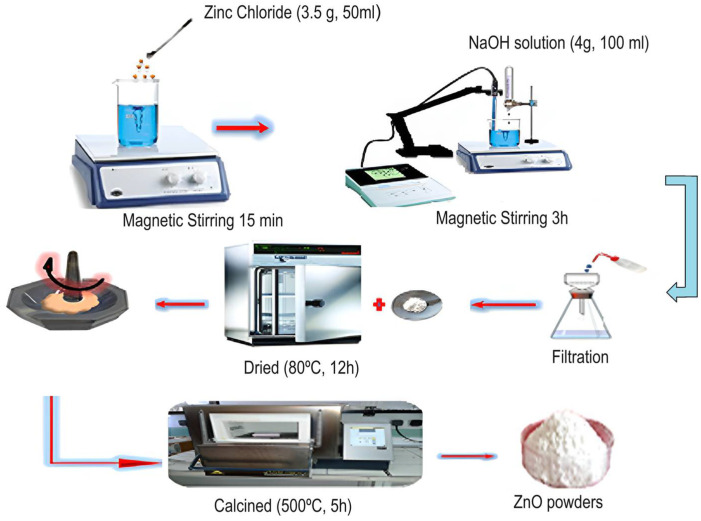
Representative scheme of the synthesis of ZnO nanoparticles via the co-precipitation process.

Figure 1 is a representative illustration of the synthesis process of ZnO nanoparticles using the co-precipitation method.

### 2.3. Material Characterization Methods

The identification of the sample structure was determined by employing the X-ray powder diffraction (XRD) technique using Panalytical (Almelo, The Netherlands), X’Pert PRO^3^. The Raman spectrum was performed at room temperature using a Spectrometer (LAB RAM HR-800) with laser excitation of 633 nm and spectral resolution of 3 cm^−1^. The N_2_ physisorption isotherms in ZnO are employed for the determination of the specific surface area, pore size distribution, and pore volume of the product. The isotherms can be measured using techniques such as the Brunauer–Emmett–Teller (BET) method using the Micromeritics BET Analyzer Gemini 2380. The photocatalytic performance of the ZnO and Co-doped ZnO nanostructures was evaluated by the degradation of MB dye under UV light using a nova^®^LIGHT TQ150 UV medium-pressure lamp. For the photocatalytic studies, 0.1 g of MB dye was dissolved in 250 mL of water and kept overnight under constant stirring to achieve a homogeneous solution. Then, 100 mg of the photocatalyst (ZnO and Co-doped ZnO) was added to 100 mL of the initial solution. After that, the resulting solution was stirred for 30 min to achieve the equilibrium of absorption and desorption between the photocatalyst and the adopted dye. Finally, the solution was irradiated with UV light for 100 min under continuous stirring. After a specific time of irradiation, the irradiated solution was collected, and its UV–vis absorption spectra were acquired for further investigation. UV−vis absorption spectra measurements were acquired on Shimadzu UV-2450 double-beam spectrometers with a 1 cm quartz cuvette over the range of 200−800 nm.

The photocatalytic efficiency for the degradation of the MB dye was calculated using the following expression:

(1) $$D\left(\%\right)\;=\;\frac{\left(C_{0}-C\right)}{C_{0}}\times 100$$

where $C_{0}$ is the initial concentration of MB and $C_{t}$ is the concentration of MB at different irradiation times.

### 2.4. Steady-State and Time-Resolved Fluorescence

Steady-state measurements were taken with Horiba–Jobin–Ivon SPEX Fluorolog 3–22 spectrometers; the spectra were corrected for the wavelength response of the system. Fluorescence lifetimes were collected using a Becker and Hickl (GmbH) DCS-120 Confocal FLIM System described elsewhere [39]. The excitation source is a picosecond diode laser of 375 nm wavelength (bh BDL series lasers) working in a pulsed mode (repetition rate: 50 MHz). The total laser power at the composition was set to 40% of the maximum value, and the collected emission passed through a long-pass filter 390 LP and bandpass filter ET642/80. The decay curves were fitted using the maximum-likelihood algorithm (or maximum-likelihood estimation, MLE) fitting method.

## 3. Results and Discussion

### 3.1. Structural and Morphological Analysis

**Figure 2:**
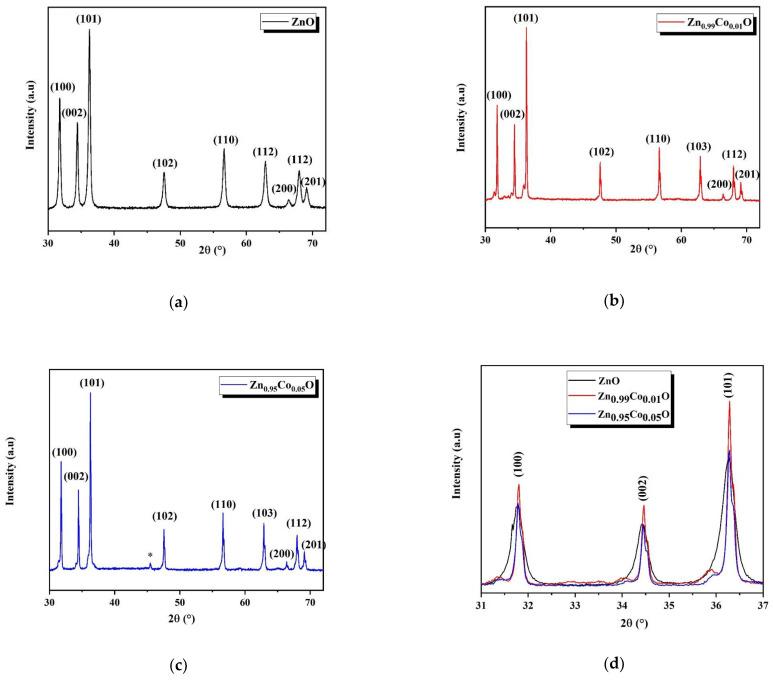
XRD pattern of (**a**) ZnO; (**b**) Zn_0.99_Co_0.01_O; (**c**) Zn_0.95_Co_0.05_O nanoparticles; (**d**) presents the shift of the diffraction peaks with increasing Co concentration.

The analysis of the XRD diffraction patterns of both ZnO and Co-doped ZnO nanoparticles was carried out to determine the crystal phases, as presented in Figure 2a–c. All the observed peaks closely match the standard JCPDS card No. 36.1541 [14], confirming the formation of a wurtzite hexagonal crystal phase in each composition.

The XRD patterns associated with the Zn_0.95_Co_0.05_O composition revealed the appearance of an additional low-intensity peak located at 45° (referenced in Figure 2c by the symbol: *), which is associated with the ZnCo_2_O_4_ cubic spinel phase. This secondary phase may be attributed to the low polarity of the ions, which reduces the interaction between precursor ions and the surface of the ZnO. Another possible reason for the formation of this secondary phase is the solubility limit of cobalt in the ZnO system [14,33].

In Figure 2d, it can be observed that the intensity of the diffraction peaks rises with the introduction of Co into the ZnO host lattice, which indicates an enhancement in the crystalline quality of ZnO nanostructures. Notably, the diffraction peaks shift towards more elevated angles with the increased Co concentration, suggesting the effective integration of Co into the ZnO host lattice. 

With respect to the hexagonal structure, the volume of the unit cell (V) for each composition is experimentally calculated according to the relationships that follow:

(2) $$V\;=\;\frac{\sqrt{3}}{2}a^{2}c$$

where *a* and *c* denote the lattice parameters and are expressed by:


(3) $$a\;=\;\frac{\lambda }{\sqrt{3}\sin\;\theta_{\left(100\right)}}$$

(4) $$c\;=\;\frac{\lambda }{\sin\theta_{\left(002\right)}}$$

The measured structural parameters are summarized in Table 1. The decline of the lattice parameters emanates from the continuing replacement of Co^2+^ doping ions (0.058 nm) by Zn^2+^ initial ions (0.074 nm) with greater ionic radius.

The average crystallite size of the compositions under study is experimentally assessed following Scherrer’s equation [14]:

(5) $$D\;=\;\frac{0.9\lambda }{\beta \;\cos\theta }$$

(6) $$\delta \;=\;\frac{1}{D^{2}}$$

(7) $$\varepsilon \;=\;\frac{\beta }{4\;\tan\theta }$$

where *λ* denotes the wavelength of the X-ray used (1.5406 Å), *β* represents the angular peak width at half-maximum in radian along (101) plane, and *θ* is Bragg’s diffraction angle.

Table 2 lists the average crystallite size, the dislocation density, and the strains. An increase in the crystallite size was revealed with higher cobalt doping concentration. Moreover, it is noticed that the dislocation density and the strains in the prepared compositions decrease with the cobalt doping content. This indicates an enhancement of the crystalline quality as cobalt is incorporated into the ZnO structure. Furthermore, Table 2 exhibits that the Zn_0.99_Co_0.01_O composition shows a larger crystallite size and reduced strains, supporting the idea that 1% of cobalt concentration is the optimal amount for achieving better structural properties.

**Figure 3:**
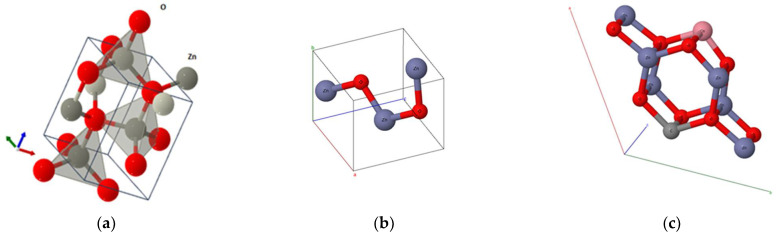
ZnO structure (**a**) before optimization, (**b**) after optimization, and (**c**) after optimization with Co doping.

The optimized geometry of ZnO bulk was achieved through the application of density functional theory (DFT) with the B3LYP hybrid functional, employing the CRYSTAL17 code. Both initial and final optimized structures are illustrated in Figure 3.

The calculated structural parameters are summarized in Table 1. Indeed, the deviation from the experimental values is roughly 1%, which indicates a good accordance between the predicted (DFT) and experimental data.

**Figure 4:**
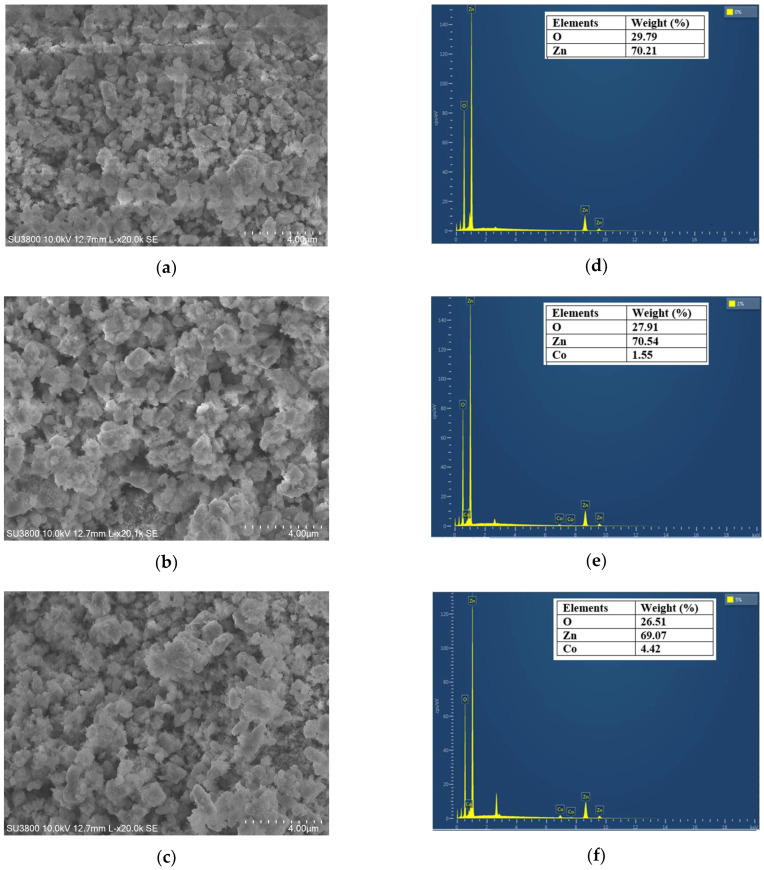
SEM images of (**a**) ZnO; (**b**) Zn_0.99_Co_0.01_O; and (**c**) Zn_0.95_Co_0.05_O, and EDX images of (**d**) ZnO; (**e**) Zn_0.99_Co_0.01_O; and (**f**) Zn_0.95_Co_0.05_O nanoparticles.

The morphology of ZnO and Co-doped ZnO samples was studied using scanning electron microscopy (SEM), as shown in Figure 4. The SEM images reveal the agglomeration of smaller spherical nanoparticles homogeneously distributed in the investigated samples. The Co doping effect increases the average particle size (see Figure 4a–c). The EDX technique was used to identify the elements (Zn, O, and Co) and their weight percentages in the analyzed compositions. The obtained EDX images were displayed in Figure 4d–f. Only zinc and oxygen were detected in the ZnO sample, which proves the higher purity of this composition. The EDX results of Zn_0.99_Co_0.01_O and Zn_0.95_Co_0.05_O reveal the appearance of additional peaks related to the Co doping element. Furthermore, the augmentation of the intensity of cobalt peaks with increasing the percentage of doping confirms the effective incorporation of the Co element into the ZnO host matrix.

### 3.2. Vibrational Properties

The impact of introducing impurities on the lattice vibrational properties of synthesized ZnO compositions and the examination of the microscopic structure by identifying the structural defects and the lattice disorder were explored using Raman scattering spectroscopy [40]. The primary goal of this analysis was to confirm the high crystallinity of the examined compositions and to detect any secondary phases that might form after Co doping. Raman spectroscopy is preferred for this purpose as it is more sensitive to material composition than XRD [34]. 

Based on the group theory, the hexagonal phase of ZnO is classified under the space group P63mc, featuring a unit cell comprising four atoms. At the Γ-point of the Brillouin zone, group theory predicts irreducible representations for the optical phonons.


(8) $$ℾ\;=\;1A_{1}+2B_{1}+1E_{1}+2E_{2}$$

**Figure 5:**
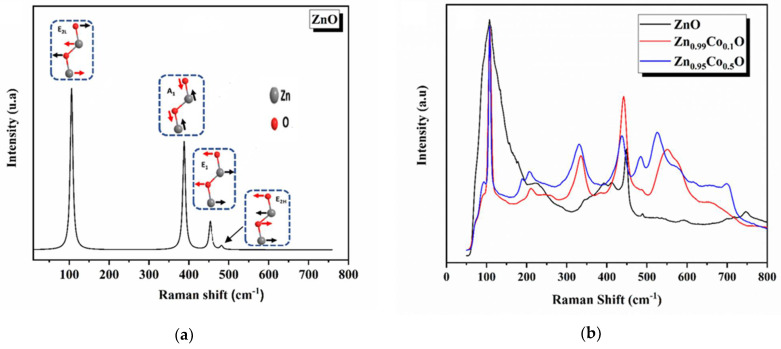
(**a**) The theoretical of ZnO and (**b**) experimental Raman spectra of ZnO and Co-doped ZnO nanoparticles. The red arrows specify the dominant Zn ions displacements.

Figure 5 displays the theoretical and experimental Raman spectra. The calculated frequencies and their assigned modes are reported in Table 3.

At room temperature, the ZnO Raman spectrum exhibits two peaks at 105 cm^−1^ and 481 cm^−1^, which are assigned to the E_2H_ and E_2L_ Raman active modes in the hexagonal structure. Although the E_2H_ mode corresponds to the oxygen (O) atoms vibrations, the E_2L_ mode is ascribed to the vibration of the zinc (Zn) sublattice. This observation accords well with those reported in previous studies [41]. The strong intensity of the E_2H_ mode indicates the good crystallinity of the ZnO nanoparticles.

The bands observed at 388 cm^−1^ and 453 cm^−1^ are assigned to the A_1_ and E_1_ modes, respectively. The agreement between the calculated and experimental findings is deemed acceptable for the E_2H_ mode, while the experimental frequency of the E_2L_ mode is slightly lower than the simulated value. This difference may be attributed to structural defects, as previous studies have revealed that tensile stress in the hexagonal phase can affect the E_2_ phonon wavenumber [41]. The observed Raman peak shift is also consistent with the compressive strain measured from XRD results. 

On the other hand, for the infrared (IR) active optical phonon modes, the calculated spectrum of wurtzite ZnO exhibits two peaks in the range of 0 to 900 cm^−1^. The characteristic bands are observed at 388 cm^−1^ and 453 cm^−1^, essentially attributed to the transverse optical (TO) phonons of the A_1_ and E_1_ modes, respectively. The intense peak is defined as the ZnO stretching vibration. The E_1_(TO) and A_1_(TO) modes provide information about the strength of the polar lattice bonds.

The experimental Raman spectra of both ZnO and Co-doped ZnO samples exhibit peak positions that align well with literature data, confirming the formation of hexagonal-structured ZnO [41,42]. In the case of the ZnO, the band at around 150 cm^−1^ could correspond to the E_2L_ mode, while the strongest peak, at approximately 440 cm^−1^, is ascribed to the E_2H_ vibrational mode, representing the characteristic mode of ZnO wurtzite structure. The existence of the characteristic mode E_2H_ in all the samples indicates the high crystalline quality of the prepared compositions. Moreover, under the influence of Co doping, in the non-polar optical phonon E_2H_ mode, the ZnO was found to change in intensity. This shift is a result of structural defects and local lattice distortion caused by the addition of Co doping elements into the ZnO host matrix [34], and it is associated with the change in the band structure of the ZnO system. The detected shift correlates with modifications observed in the optical absorption spectra of our studied samples, as previously discussed in our research [14]. The Raman peaks located at approximately 340 cm^−1^, 392 cm^−1^ and 413 cm^−1^ are accredited to the E_2H_-E_2L_, A_1_(TO), E_1_(TO), respectively [40]. An increase in the intensity of the peaks with Co addition to the ZnO host lattice confirms the effective incorporation of cobalt into ZnO [41]. This peak is related to the vibrational states of Co-O-Co chain complexes [42]. Another vibrational mode at 618 cm^−1^, observed for the Zn_0.95_Co_0.05_O composition, is the Co doping effect and is attributed to the F_2g_ vibrational mode characteristic of ZnCo_2_O_4_. The peak at around 572 cm^−1^ is due to the A_1_(LO) mode and is linked to the structural disorder in the host lattice, including oxygen vacancy (V_O_), zinc interstitial (Zn_i_), and their complexes [42,43,44,45]. An observed change in the intensity of this peak, particularly for the Zn_0.95_Co_0.05_O composition, suggests an increased concentration of oxygen vacancies in this specific composition due to cobalt addition. Additionally, the Raman spectra of Co-doped ZnO samples reveal an additional peak at 550 cm^−1^, indicating effective Co doping. This peak is attributed to the vibrational mode of Co-O-Co chain complexes [46]. Another vibrational mode located at 618 cm^−1^, observed for the Zn_0.95_Co_0.05_O composition, is primarily associated with the Co doping effect and accredited to the F_2g_ vibrational mode characteristic of ZnCo_2_O_4_ [47].

### 3.3. Time-Resolved Photoluminescence Measurements

Time-resolved photoluminescence (TRPL) measurements are commonly employed to assess the quality of ZnO measurements. It provides valuable information about the exciton lifetime, which reflects the efficiency of the radiative recombination and serves as an important parameter for characterizing a material for specific applications. Generally, the degeneration of ZnO emission is contemplated as a multiexponential process with both short and long components. In this context, ZnO nanostructures may exhibit either a single-exponential or a bi-exponential decay, both of which have already been presented in the ZnO matrix [48]. Bi-exponential decay typically involves a fast decay component linked to the hole-electron recombination and a slow decay associated with the radiative lifetime of the free exciton. The fast process is often attributed to defects. In our study, all samples exhibited a bi-exponential decay trend.

**Figure 6:**
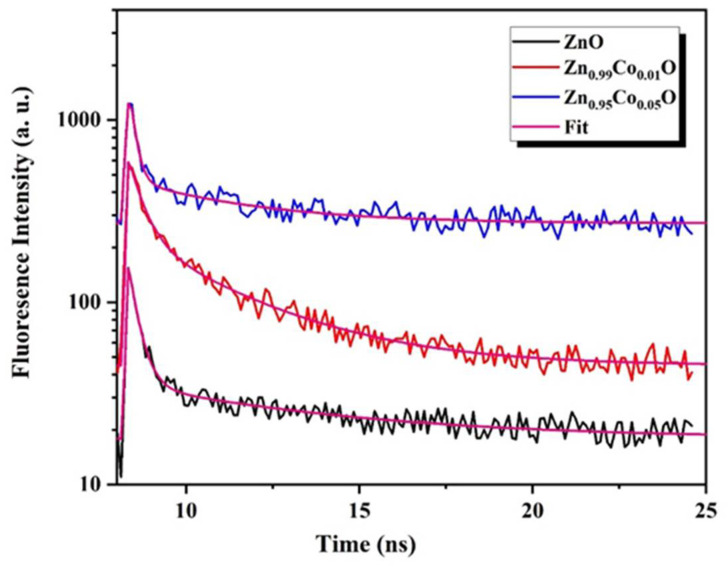
Time-resolved photoluminescence decay curves of ZnO and Co-doped ZnO nanoparticles.

As clearly observed in Figure 6, the decay curves are well fitted to the bi-exponential model expressed by the following relation [49]:


(9) $$I\left(t\right)\;=\;A_{1}exp\left(\frac{-t}{\tau_{1}}\right)+A_{2}exp\left(\frac{-t}{\tau_{2}}\right)$$

In this expression, $I\left(t\right)$ represents the time-dependent luminescence intensity, $\tau_{1}$ and $\tau_{2}$ denote the decay times of the fast and the slow decay components, respectively; $A_{1}$ and $A_{2}$ represent the relative amplitudes of the fast and slow decay components, respectively.

Using the fitting parameters in Equation (9), the effective intensity average lifetime can be evaluated based on the following equation:


(10) $$\tau_{eff}\;=\;\frac{A_{1}\tau_{1}^{2}+A_{2}\tau_{2}^{2}}{A_{1}\tau_{1}+A_{2}\tau_{2}}$$

The introduction of Co doping has a significant impact on the decay time components and their respective relative intensities, as shown in Table 4. It is evident that the effective intensity average lifetime increases under the influence of Co doping, indicating an enhancement in radiative recombination [49]. This increase is initially observed for the Zn_0.99_Co_0.01_O composition and subsequently decreases for the Zn_0.95_Co_0.05_O composition. This phenomenon is likely attributed to the creation of intermediate states introduced by Co doping into the ZnO host matrix, which initially hampers charge carrier recombination. However, beyond a certain critical Co doping concentration (1%), the overabundance of doping can act as a recombination center rather than suppressing recombination, paradoxically resulting in the opposite effect. As a result, the photoluminescence (PL) lifetime decreases beyond this critical doping level [1]. Al-Namshah et al. [3] similarly observed an enhancement in photoactivity response up to a certain critical Co concentration in their study of Co-doped ZnO nanostructures.

Based on the findings of this study, we conclude that the optimal cobalt doping concentration is 1%, aligning with the highest recorded lifetime of 7.11 ns. This finding aligns with the observations made by Mondal et al. [48], who indicated that a decrease in carrier lifetime leads to a reduction in the photodegradation response, often associated with defect-related recombination induced by oxygen vacancies.

The increment of the PL lifetime for Zn_0.99_Co_0.01_O composition reflects an easiness of the charge separation ability in the Co-doped ZnO nanoparticles, which highlights the potential of this approach for the degradation of organic pollutants applications [50].

### 3.4. BET and BJH Studies

Understanding the porosity and surface area of nanomaterials plays a fundamental role in assessing their functionality and quality. Notably, materials with a higher surface area provide a multitude of active sites, therefore contributing significantly to improved photocatalytic performance. In this context, we evaluated the textural properties of the synthesized ZnO samples through N_2_ physisorption measurements. To accomplish this, the prepared compositions were subjected to a heating process at 200 °C for 1 h, followed by vacuum degassing to eliminate contaminants such as absorbed water and other gases. Subsequently, the compositions were subjected to analysis through exposure to nitrogen gas at a constant temperature of 77 K. The applied pressure varied until reaching equilibrium.

**Figure 7:**
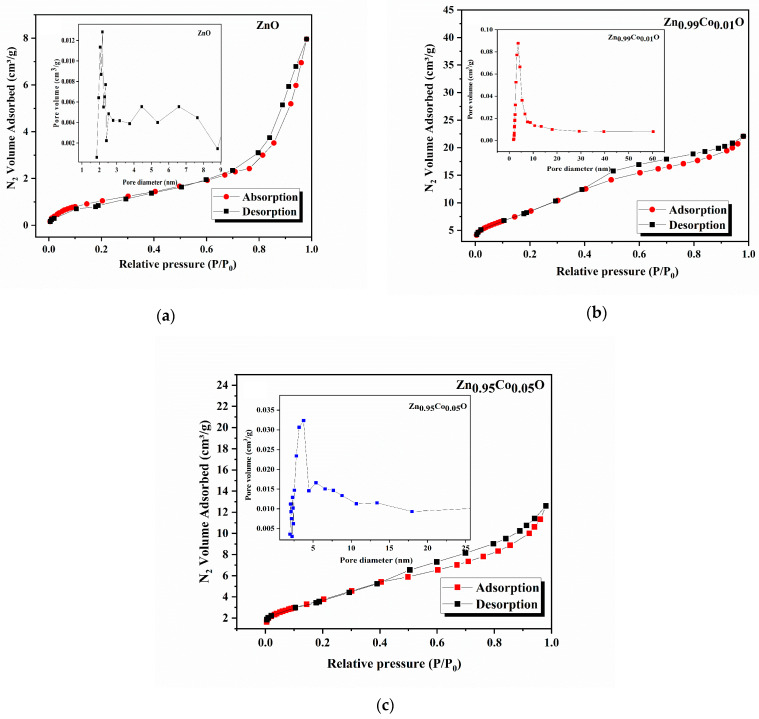
Adsorption–desorption of (**a**) ZnO, (**b**) Zn_0.99_Co_0.01_O, and (**c**) Zn_0.95_Co_0.05_O. The inset presents the BJH pore distribution of each sample.

The N_2_ adsorption–desorption isotherms for the ZnO and Co-doped ZnO samples are presented in Figure 7. Additionally, the insets in Figure 7 present the BJH pore distribution of each sample. The specific surface area was evaluated from the BET plot according to the following BET equation [17]:


(11) $$\frac{1}{Q\left[\left(\frac{P_{0}}{P}\right)-1\right]}\;=\;\frac{c-1}{Q_{m}c}\left(\frac{P}{P_{0}}\right)+\frac{1}{Q_{m}c}$$

In this expression, *P*_0_ and *P* correspond to the saturation and equilibrium pressures of adsorbate, *Q_m_* denotes the quantity of monolayer adsorbed gas, *Q* signifies the quantity of adsorbed gas, and *c* represents the BET constant. It can obviously be seen that all the samples exhibit the characteristic type IV isotherms, according to the IUPAC classification [17].

Table 5 lists the BET surface area values for the ZnO, Zn_0.99_Co_0.01_O, and Zn_0.95_Co_0.05_O compositions, which were determined as 4.13, 51.11, and 13.68 (m^2^/g), respectively. In addition, it can be observed from the BET surface area results that the cobalt doping increases the surface area of the ZnO system, which may lead to augmented photocatalytic performance. As observed in this table, Co doping enhances the BJH adsorption and desorption average pores’ width of the ZnO system.

One possible reason for this phenomenon could be that metal oxide particles are blocking the pores in the doped compositions. These particles may exist both inside the cobalt pores and on its surface at the same time. Another explanation might be that cobalt influences the porosity of the ZnO system. As shown in Table 5, our results align with the findings reported by Patehkhor et al. [51], who studied TiO2 ZnO/CS Gr nanocomposites made using an ultrasound-assisted fabrication method.

### 3.5. Photocatalytic Properties

**Figure 8:**
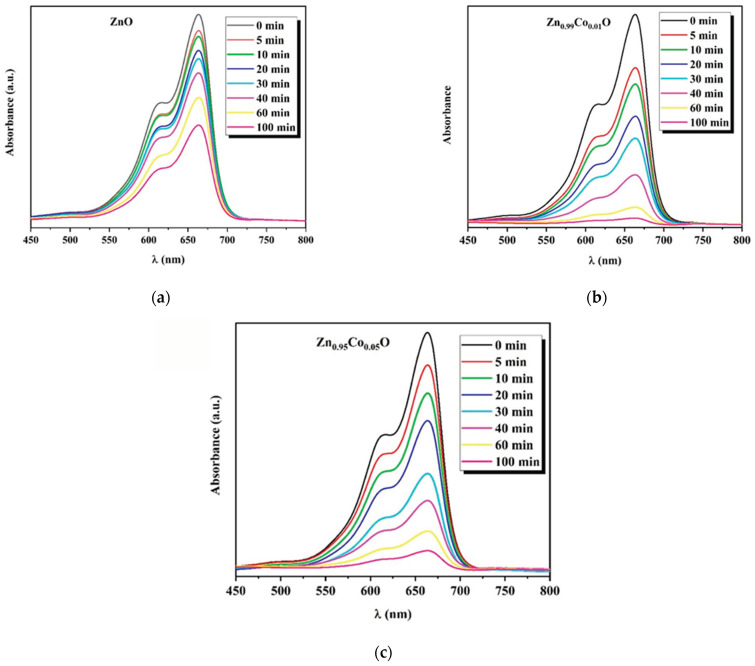
UV–vis spectra of MB degradation for (**a**) ZnO, (**b**) Zn_0.99_Co_0.01_O, and (**c**) Zn_0.95_Co_0.05_O nanoparticles.

The photocatalytic performance of ZnO and Co-doped ZnO with different doping concentrations (1% and 5%) was investigated by decomposing MB in an aqueous solution irradiated with UV light that is selected as a representative organic pollutant in this work. The samples were taken for 30 min in a dark setting for the achievement of the adsorption/desorption equilibrium. To start the photocatalysis, the lamp was turned on for a total of 100 min of reaction time. The change in the UV–vis absorption of the MB solution for the fabricated compositions under UV irradiation is presented in Figure 8.

The characteristic MB absorption peak located around 665 nm gradually decreases with the UV light irradiation time, indicating the progressive degradation of the dye molecules. Furthermore, a decrease in the absorption was clearly seen earlier, mostly in the case of Co-doped ZnO compositions, indicating the effective photocatalytic degradation response of the ZnO system under the cobalt doping effect. 

To gain a deeper insight into the mechanisms involved in the degradation of methylene blue (MB) through photocatalysis reaction, we present insightful interpretations. First, the dye attaches to the surface of ZnO nanostructures. When these ZnO nanostructures with MB attached are exposed to UV light, it initiates the creation of an electron–hole pair within the ZnO material, as shown in Equation (12). These photogenerated electrons in the conduction band of ZnO nanostructures interact with the oxygen molecules that have been adsorbed to produce superoxide anion radicals $\left(.O_{2}^{-}\right)$ as depicted in Equation (13). Then, the holes created in the ZnO valence band react with surface hydroxyl groups, resulting in reactive hydroxyl radicals (.OH) as indicated by Equation (14). These holes, formed through photoexcitation, can also cause water molecules in the solution to dissolve and form radicals (Equation (15)). The highly reactive hydroxyl radicals (.OH) and superoxide radicals $\left(.O_{2}^{-}\right)$ interact with MB dye adsorbed on ZnO nanostructures, leading to its degradation and decolorization, ultimately resulting in a colorless form (Equations (16) and (17)). 

According to the literature, the proposed mechanism for the efficient degradation of MB under UV light irradiation in the ZnO nanostructures can be finally defined by the following reaction [8,17,52]:


(12) $$ZnO+hv\to e^{-}\left(CB\right)+h^{+}\left(VB\right)$$

(13) $$e^{-}+O_{2}\to \;{.O}^{2-}$$

(14) $$h^{+}+{OH}^{-}\to \;.OH$$

(15) $$h^{+}+H_{2}O\to H^{+}+.OH$$

(16) .OH+organic dye→degradation product

(17) $$.O_{2}^{-}+organic\;dye\to degradation\;product$$

For further investigation, the Langmuir–Hinshelwood (L-H) model was employed to evaluate the degradation rate constant of MB dye [40]. This model is expressed by the following relationship:


(18) $$ln\left(\frac{c_{0}}{c}\right)\;=\;k\;t$$

**Figure 9:**
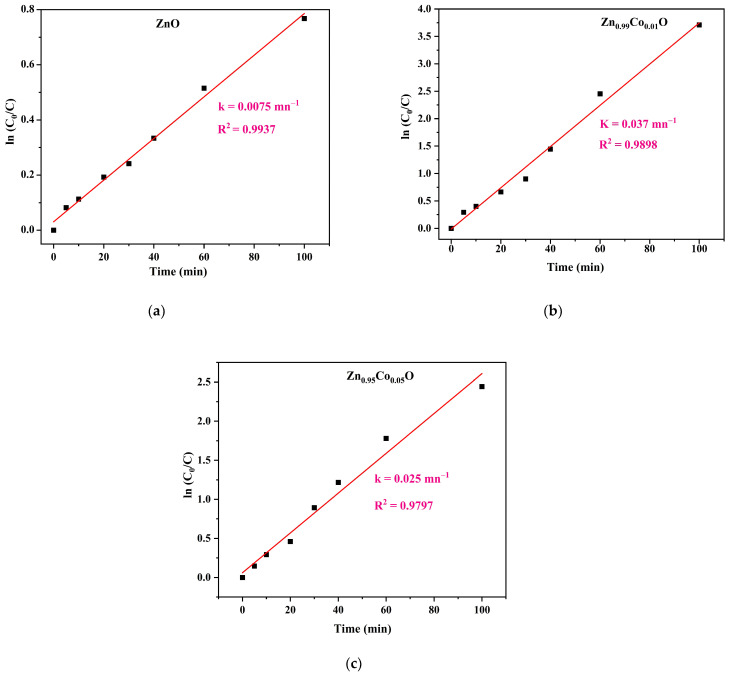
Pseudo-first kinetic plots of (**a**) ZnO, (**b**) Zn_0.99_Co_0.01_O, and (**c**) Zn_0.95_Co_0.05_O nanoparticles.

In this expression, *k* describes the first-order rate constant, *c*_0_ denotes the initial MB concentration, and *c* presents the concentration of MB after illumination time *t*. The pseudo-first-order rate was determined from the slope of $ln\left(\frac{c_{0}}{c}\right)$ versus irradiation time, as observed in Figure 9.

All the compositions exhibit nearly linear curves, suggesting that the photocatalytic degradation of MB follows pseudo-first-order kinetics. The calculated rate constants initially increase from 0.0075 min^−1^ for ZnO to 0.037 min^−1^ for Zn_0.99_Co_0.01_O before decreasing to 0.020 min^−1^ for Zn_0.095_Co_0.05_O sample. This pattern demonstrates that Co doping enhances the photocatalytic performance of the ZnO nanoparticles. In particular, a higher first-order rate constant is associated with a more significant photocatalytic activity [19]. Hence, the Zn_0.99_Co_0.01_O sample offered the highest kinetics constant value, demonstrating again the higher photocatalytic activity of this composition than the other samples [31]. 

**Figure 10:**
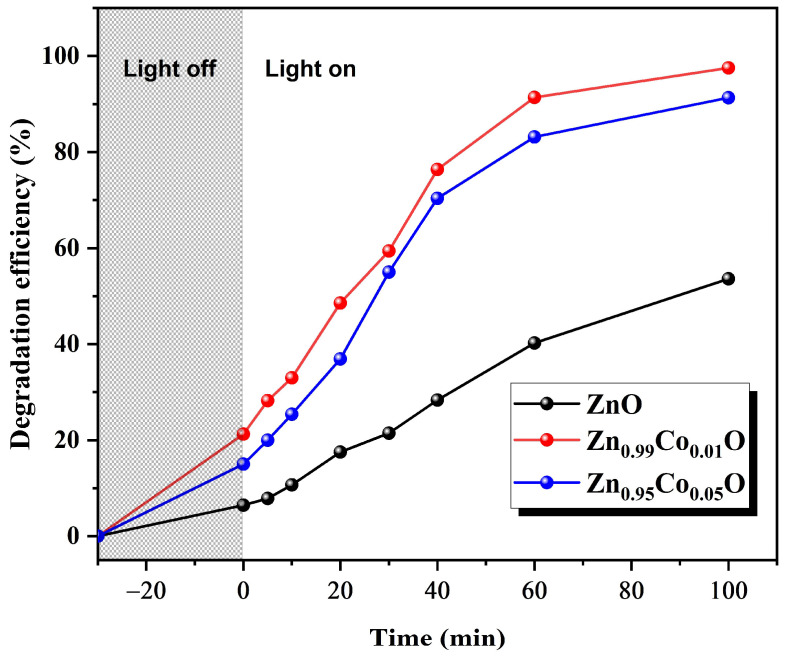
Plots of degradation of MB as a function of time of ZnO, Zn_0.99_Co_0.01_O, and Zn_0.95_Co_0.05_O nanoparticles.

The photocatalytic performance of ZnO and Co-doped ZnO with different doping concentrations (1% and 5%) was investigated by decomposing MB in an aqueous solution irradiated with UV light that is selected as a representative organic pollutant in this research paper. The samples were taken for 30 min in a dark setting for the achievement of the adsorption/desorption equilibrium. To start the photocatalysis, the lamp was turned on for a total of 100 min of reaction time. Figure 10 illustrates the enhancement of the photocatalytic activity under Co doping. Zn_0.99_Co_0.01_O composition displays the highest photocatalytic activity, with 97% degradation of MB compared to 87% for Zn_0.95_Co_0.05_O composition, which in turn is higher than 53% for ZnO nanoparticles. 

Several factors contribute to this improvement. The enhancement in photocatalytic activity when incorporating cobalt (Co) as a dopant into the ZnO host lattice can be attributed to various factors. First, the notable improvement in the degradation of MB achieved with 1% Co doping concentration can be attributed to the increased surface area and enhanced crystallinity. These effects promote the efficient separation of electron–hole pairs, facilitating the separation of photogenerated charge carriers within the ZnO system, ultimately leading to an augmentation in photocatalytic efficiency. Materials with large surface areas and higher crystallinity provide more active sites for photogenerated charge carriers, ultimately improving photocatalytic activity. Additionally, the enhanced photocatalytic activity of the Zn_0.99_Co_0.01_O sample is likely to emanate from the long lifetime of the exciton, which is an observation consistent with those found in other research works [17]. 

Second, the minor decline in the degradation of the organic pollutant, particularly in the Zn_0.95_Co_0.05_O sample, can be attributed to the potential emergence of a secondary phase, ZnCo_2_O_4_. This secondary phase reduces the effective surface-active sites. The presence of this new phase on the ZnO surface hinders the separation and transportation of photogenerated charge carriers, resulting in a decrease in photocatalytic activity. Similar findings have been reported by Lu et al. [52]. 

This decrease emanates from the change in the morphology. It is broadly known that several factors strongly affect the photocatalytic features of metal oxide nanostructures. These include morphology, crystallinity, surface area, porosity, etc. [26,40]. Within this framework, the change in the structure, shape, and surface morphology of the material is pondered as latent factors that strongly affect the efficacy of the ensuing degradation. Indeed, Zeng et al. [53] have hydrothermally prepared single crystalline ZnO nanodisks and nanowires via the hydrothermal technique and found that ZnO nanodisks with a high population of (0001) facets exhibit the best catalytic activity for photodegradation of rhodamine B dye as compared to ZnO nanowires. ZnO nanodisks with a high population of (0001) facets exhibited the best catalytic activity for the photodegradation of rhodamine B dye compared to ZnO nanowires.

Similarly, Kuriakose et al. [31] reported the preparation of Co-doped ZnO nanodisks and nanorods through a simple wet chemical process. They conducted a comparison of the photocatalytic performance of these two different morphologies by degrading an aqueous MB solution under sunlight irradiation. Their findings showed an enhancement in the photocatalytic activity of both ZnO nanodisks and nanorods under the Co addition. Importantly, nanodisks exhibited superior photocatalytic activity compared to other morphologies. They attributed this improvement to the combined effects of the increased surface area of ZnO nanodisks and enhanced charge separation efficiency, which impedes the recombination of the photogenerated charge carriers.

Such enhancement of the photocatalytic activity with the addition of cobalt element into the ZnO host lattice has also been documented by many researchers. As summarized in Table 6, we conducted a comparative study of the performance of our findings with those reported by other research works on Co-doped ZnO nanostructures with diverse shapes and morphologies in terms of their dye photodegradation efficiency [54,55,56,57,58,59].

As can be illustrated, the photocatalytic response of Co-doped ZnO is intensely contingent on the structure, shape, and surface morphology of the nanostructures generated by several methods. Compared to the reported studies in the literature, our material exhibited a higher removal efficiency equal to 97% and achieved this result in a shorter period of degradation time. Furthermore, the higher photocatalytic activity of Zn_0.99_Co_0.01_O nanoparticles suggests that the newly prepared photocatalyst has the potential for application in environmental contexts.

### 3.6. Mechanical Properties

Calculating the elastic properties represents a substantial tool to provide information about the mechanical behavior of each composition of metal oxide nanostructures [60]. For each optimized structure, the parameters of bulk modulus K, shear modulus G, Young’s modulus E, and Poisson’s ratio were calculated. Regarding the bulk modulus K, it reveals the reduction in the volume following an increase in pressure, while the shear modulus G presents the ratio of shear stress to the shear strain. Furthermore, Young’s modulus E and Poisson’s ratio ν are utilized to assess the rigidity and ability of our metal oxide to enlarge in perpendicular directions to the compression direction.

**Figure 11:**
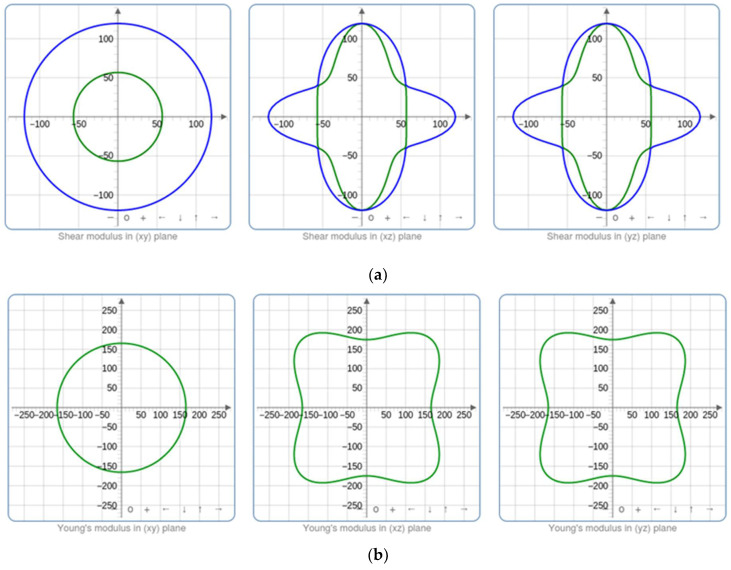

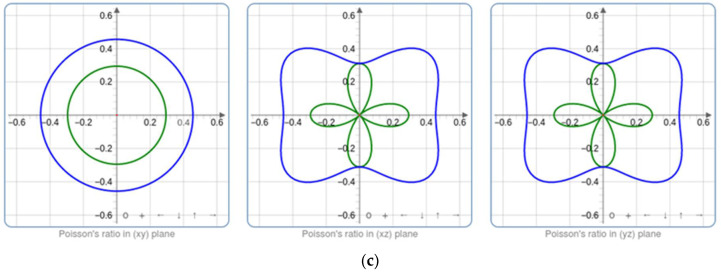
The variation in the (**a**) shear modulus, (**b**) Young’s modulus, and (**c**) Poisson’s ratio for the ZnO sample.

Figure 11 illustrates how the material’s compressibility varies at various points within the crystal lattice due to the spatial dependence of the elastic characteristics. Higher bulk modulus values imply less compressible zones, while lower bulk modulus values suggest more compressible parts. However, Young’s modulus reveals zones with various stiffness values. Greater stiffness suggests greater resistance to deformation, which is associated with higher Young’s modulus regions. Based on these findings, it can be inferred that the material may exhibit varied mechanical responses along distinct crystallographic orientations. This variability can be attributed to the presence of impurities within our structures, leading to localized differences in Young’s modulus and shear modulus. 

The bulk modulus to shear modulus ratio (K/G) is a key metric for evaluating a material’s ductility or brittleness. A lower K/G ratio signifies higher ductility, indicating the material can deform significantly before fracturing. Conversely, a higher K/G ratio suggests increased brittleness, with the material prone to fracturing with minimal deformation. This ratio provides essential insights into the mechanical behavior of materials, aiding in predictions of their performance under different loading conditions. These elastic parameters are chosen in accordance with the relationships mentioned in the study of Elhamra et al. [61]. Table 7 provides a summary of the outcomes.

In the present work, K/G values are significantly greater than 1, which increases with Co doping, suggesting that brittleness is favored. This is because the material’s resistance to shear deformation (G) is much higher compared to its resistance to volume changes (K). This phenomenon can be attributed to the strong, directional bonds in ZnCoO, making it challenging for dislocations to move and accommodate deformation. 

In summary, the determination of the elastic properties of ZnO, especially Co-doped ZnO, is crucial for understanding and optimizing its photocatalytic performance. These properties play a pivotal role not only in enhancing photocatalytic activity through strategically introduced dopants such as Co but also in ensuring recyclability. Additionally, the impact of a higher surface area on reactivity is noteworthy, with well-crystallized structures contributing to stability and elastic properties influencing charge carrier mobility. A comprehensive understanding and manipulation of these properties are imperative for tailoring ZnO-based photocatalysts, facilitating the design of more efficient and durable materials for applications such as environmental remediation and solar energy conversion.

## 4. Conclusions

In this study, we conducted the synthesis of nanopowdered Zn_1−x_Co_x_O (0 ≤ x ≤ 0.05) samples by varying the cobalt doping concentration through a chemical co-precipitation approach. We characterized the photocatalysts using X-ray diffraction, SEM, and Raman spectroscopy to explore the influence of cobalt doping on the properties of ZnO nanostructures. The structural and vibrational properties were further investigated through DFT calculation. A good agreement between experimental findings and DFT calculation for the Co-doped ZnO system is achieved. Structural analysis confirmed that all samples exhibited well-crystallized hexagonal wurtzite structures of the ZnO phase. The crystallite size increased from 26.5 nm for ZnO to 53.9 nm for Zn_0.99_Co_0.01_O and then slightly decreased (51.6 nm) for Zn_0.95_Co_0.05_O nanoparticles. The same evolution of agglomerated spherical nanoparticles was observed with SEM images. Vibrational properties revealed the hexagonal wurtzite structure for all samples, along with an additional Raman peak indicative of the F_2g_ vibrational mode characteristic of ZnCo_2_O_4_. This mode affirmed the successful incorporation of cobalt doping into the ZnO lattice and indicated the formation of the secondary phase ZnCo_2_O_4_ for 5% cobalt doping concentration. 

Additionally, DFT calculation allowed the assessment of the cobalt doping’s impact on the elastic properties of ZnO, resulting in enhanced mechanical properties and recyclability as the Co doping increased. On the other hand, the studied compositions showed a relevant efficiency as photocatalysts in the degradation of MB dye under UV light irradiation. Accelerated degradation was attributed to key factors such as the material’s extended photoluminescence lifetime, a slower rate of photogenerated charge carrier recombination, and increased surface area. 

The obtained results highlighted the superior efficiency of 97% for the photocatalyst with a Zn_0.99_Co_0.01_O composition, indicating optimal photocatalytic activity with exceptional efficiency compared to that reported in the literature. This study underscores the improvement of the ZnO system’s structure and photocatalytic characteristics, particularly for lower cobalt doping concentrations (1%). The developed compositions have demonstrated their potential as promising materials for more efficient, stable, and durable photocatalysts for environmental applications owing to their enhanced photocatalytic and mechanical performance.

## Figures and Tables

**Table 1:** Comparison between the experimental structural parameters of ZnO and Co-doped ZnO nanoparticles and theoretical values obtained from DFT calculation.

	Lattice Constants of ZnO Nanoparticles Obtained from Experimental Data				Lattice Constants of ZnO Nanoparticles Obtained from DFT Calculation		
Compositions	a (Å)	c (Å)	$V\;({Å}^{3})$	Compositions	a (Å)	c (Å)	$V\;({Å}^{3})$
ZnO	3.2540	5.2123	47.7970	ZnO	3.2667	5.2615	48.6250
Zn_0.99_Co_0.01_O	3.2512	5.2072	47.6678	Zn_0.973_Co_0.027_O	3.2521	5.2345	47.9425
Zn_0.95_Co_0.05_O	3.2499	5.2058	47.6168	Zn_0.945_Co_0.055_O	3.2478	5.2301	47.7756

**Table 2:** Values of crystallite size, dislocation density, and strains of ZnO and Co-doped ZnO.

Compositions	*D* (nm)	*δ* (*line.nm*^−2^)	*ε*
ZnO	26.5	0.00124	0.0040
Zn_0.99_Co_0.01_O	53.9	0.00034	0.0019
Zn_0.95_Co_0.05_O	51.6	0.00037	0.0021

**Table 3:** Detailed assignment of theoretically computed IR and Raman vibrations of ZnO.

Frequency (cm^−1^)	Symmetry	IR	Raman
105.87	E_2_	I	A
280.07	B	I	I
388.07	A	A	A
453.81	E_1_	A	A
481.82	E_2_	I	A
583.34	B	I	I

**Table 4:** The PL lifetime parameters derived from kinetic analysis of emission decay of ZnO and Co-doped ZnO.

Compositions	*A*_1_ (%)	*τ*_1_ (*ns*)	*A*_2_ (%)	*τ*_2_ (*ns*)	*τ_eff_* (*ns*)
ZnO	94.80	0.645	2.61	2.60	2.60
Zn_0.99_Co_0.01_O	88.98	0.29	11.05	8.90	7.11
Zn_0.95_Co_0.05_O	86.34	0.21	13.66	3.83	2.87

**Table 5:** BET surface area and BJH values for ZnO and Co-doped ZnO nanoparticles.

Compositions	Surface Area (m^2^/g)	BJH Adsorption Average Pore Width (Å)	BJH Desorption Average Pore Width (Å)
ZnO	4.13	95.207	98.765
Zn_0.99_Co_0.01_O	51.11	42.362	39.865
Zn_0.95_Co_0.05_O	13.68	59.681	58.630

**Table 6:** Comparison of photocatalytic activities of ZnO and Co-doped ZnO nanoparticles.

Type of Catalyst	Type of Dye	Light Source	Degradation Efficiency (%)	Time of Exposure
Co-doped ZnO nanorods [54]	Methyl Orange (MO)	500 W xenon lamp with 420 nm excitation	48.2	360 min
Co-doped ZnO nanorods [55]	Rhodamine B (RhB)	Under artificial solar spectrum	66.5	100 min
Co-doped ZnO nanowires [56]	Methyl Orange (MO)	Under visible light irradiation	50	360 min
Co-doped ZnO (4%) nanoparticles [57]	Congo Red	16 W UV lamp with a wavelength of excitation of 254 nm	54.1	120 min
Co-doped ZnO (4%) thin film [58]	Methylene Blue (MB)	16 W UV lamp with a wavelength of excitation of 254 nm	76.31	120 min
Co-doped ZnO (5%) nanoparticles [59]	Methylene Blue (MB)	Under visible light irradiation	65	300 min
Co-doped ZnO (5%) nanowires [27]	Methyl Orange (MO)	Under visible light irradiation	71	300 min

**Table 7:** Average properties of ZnO and Co-doped ZnO nanoparticles.

Averaging Scheme	Bulk Modulus	Young’s Modulus	Shear Modulus	Poisson’s Ratio
Voigt	K_V_ = 190.38 GPa (ZnO)	E_V_ = 220.83 GPa (ZnO)	G_V_ = 84.5 GPa (ZnO)	ν_V_ = 0.30 (ZnO)
	K_v_ = 200.54 GPa (ZnCoO)	E_v_ = 223.4 GPa (ZnCoO)	Gv = 84.98 GPa (ZnCoO)	ν_V_ = 0.31 (ZnCoO)
Reuss	K_R_ = 188.51 GPa (ZnO)	E_R_ = 200.53 GPa (ZnO)	G_R_ = 75.80 GPa (ZnO)	ν_R_ = 0.32 (ZnO)
	K_R_ = 200.22 GPa (ZnCoO)	E_R_ = 197.62 GPa (ZnCoO)	G_R_ = 73.98 GPa (ZnCoO)	ν_R_ = 0.33 (ZnCoO)
Hill	K_H_ = 189.45 GPa (ZnO)	E_H_ = 210.74 GPa (ZnO)	G_H_ = 80.15 GPa (ZnO)	ν_H_ = 0.31 (ZnO)
	K_H_ = 200.38 GPa (ZnCoO)	E_H_ = 210.61 GPa (ZnCoO)	G_H_ = 79.48 GPa (ZnCoO)	ν_H_ = 0.32 (ZnCoO)

## Data Availability

The data presented in this study are available on request.
